# Observations on Mr. Brodie's Experiments, &c.

**Published:** 1811-10

**Authors:** 


*To the Editors of the Medical and Physical Journal.*

GENTLEMEN,

I YESTERDAY met with Mr. Brodie's account of the experiments which he has made on animals, with a view to ascertain the influence of the brain on the action of the heart, and and on the generation of animal heat, as given in the Number of your Medical Journal for July last; and I have taken the liberty of troubling you with the following observations on that publication, thinking that you will not find them unworthy of a page in your valuable work.

Mr. Brodie has drawn the following conclusions from the experiments which he made.

1st. That the influence of the brain is not directly necessary to the action of the heart.

2d. That when the brain is injured or removed the action of the heart ceases, only because respiration is under its influence, and if under these circumstances respiration is artificially produced, the circulation will still continue.

3d. When the influence of the brain is cut off, the secretion of urine appears to cease, and no heat is generated; notwithstanding the functions of respiration and the circulation of the blood continue to be performed, and the usual changes in the appearances of the blood are produced in the lungs.

4th. When the air respired is colder than the natural temperature of the animal, the effect of the respiration is not to generate, but to diminish animal heat.

More than these conclusions it would be unnecessary to extract, as so late a publication can be most easily referred to; the observations which I have made on them were suggested to me by the perusal of the experiments made by Mr. Brodie, and I regret that it has not been in my power to make some additional ones.

As it appears, that notwithstanding the continuance of respiration and circulation, the heat of the animal diminishes in a proportion greater even than if it was simply dead, and as the secretions seem to be suspended at the same time, it is by no means improbable that they are very closely connected together, that they depend upon the same cause, or even that *the former is only one of the phænomena attendant upon the production of the latter*.

The heart, although not *directly* under the influence of the brain, *is so in a considerable degree*; the contrary of which it would be absurd to suppose; but it is absolutely proved, by the decline of its powers, from the time that the connection of that organ with the rest of the system is destroyed. The increase in the frequency of the pulsations, which in one or two cases took place, may be considered either as convulsive motions, of which there were many instances in the other muscles, or as more frequent and at the same time more feeble actions of the heart.

*The nervous power residing in the muscular system seems not to be so immediately dependant upon the brain, as that which*

*which appertains to the system of secretion, &c.* for long after the latter seems to be abolished, the former is abundantly manifested by the continuance of the pulsation of the heart and arteries, the evacuation of the fœces, and the movements of the voluntary muscles. Before, however, this conclusion can be fairly established, it ought to be more fully proved that the secretions are suspended; and it would be desirable to ascertain by more experiments whether or not this is the case; to see whether the digestive process could not be carried on during this state of artificial life, or whether actions, at any rate analogous to secretion, could not be excited by the application of vesicatories of different kinds, &c.

The usual changes in the blood took place in the round of the circulation, yet no effect was produced by this, either on the secretions or the generation of heat; but is it likely that no effect whatever was produced? To me it seems extremely probable *that the alteration which still took place in the blood, when it passed through the lungs, was calculated to supply a necessary stimulus to the arterial and muscular systems,* (the former of which indeed may be considered as a branch of the latter) for these were the only ones which remained in a state of activity, and on these only could the materials gained from the atmosphere be expended; for as the secretions were at an end there is reason to suppose the process of nourishment was also; and if so, the change wrought in the blood could only be to benefit the systems before mentioned.

There is another reason which induces me to think that the red blood is the natural stimulus to the arterial system, and that the changes which take place in the lungs are principally subservient to this end; and I principally found this opinion upon the phenomena which take place in consequence of suspended respiration, in which case I conceive the destruction of life proceeds from the failure of the action of the arterial system, (its proper stimulus being withheld) and not from the influence of the black blood on the brain, producing an abolition of nervous power, as has been strongly maintained of late.

If an animal lies apparently dead in consequence of obstructed respiration, what are the means resorted to in order to restore life?

We first produce an artificial respiration, by which the blood which has not as yet lost its vitality becomes converted from black to red; but although by this means a quantity of blood has been produced fit for the purposes of life, yet it is not applied to the heart, it is not in motion; in order to effect this also, we set on foot an artificial circulation, by pressing the blood from the extremities towards the heart, this this gradually displaces that, which was before stagnant in the right cavities, which entering the pulmonary system in its turn pushes forwards successively into the left cavities, first that which occupied the pulmonary veins, and afterwards that which had undergone the proper changes in the air cells; when *this* reaches the left cavities it stimulates them to action, which at first is feeble, but gradually becomes stronger.

But it may be argued, that the restoration of life is not owing to the red blood being again forced into the left cavities of the heart, but by a continuance of the process into the brain; this is possible, but I should ask, how does the circulation cease when its cessation proceeds from causes operating on the brain? I believe it is generally admitted, from the muscles of respiration first ceasing their action, and in consequence the heart failing to receive its supply of blood. Now, of course, we must suppose if the circulation were re-established, through the agency of the brain, that the actions would recommence first in the organs of respiration, which are more immediately under its influence, and then in the heart; the reverse however of this is the case, for the heart begins to pulsate before the respiration is renewed; therefore we are warranted in concluding that life began where it first ceased, that it ceased from red blood being no longer brought to the heart, and that it recommenced from that supply being restored.

A very simple experiment however would suffice to set this question at rest; for if instead of changing the air which is used in the artificial respiration, we were to continue to use the same over and over again, after the communication between the brain and the system at large has been cut off, by removing the animal's head; if the pulsations were to cease, (as they most probably would) from the circulation of black blood, then it would be proved that this cause acted on *the heart, and not on the brain.*

These, Gentlemen, are the few observations which I have been induced to make on this subject, by which it will appear that I have no wish to controvert the conclusions which Mr. Brodie has drawn; but rather to carry them further than he has, and to suggest some new experiments which might perhaps cast fresh light on this intricate subject.

I have the honour to be,

Yours, &c. &c.

J. H. J.

*Exeter, Aug.* 23.

